# Orexin A peptidergic system: comparative sleep behavior, morphology and population in brains between wild type and Alzheimer’s disease mice

**DOI:** 10.1007/s00429-021-02447-w

**Published:** 2022-01-23

**Authors:** Peng Zhao, Yaqian You, Zhe Wang, Yanjun Zhou, Gaoshang Chai, Gen Yan, Zhewu Jin, Qing Wang, Hongxu Sun

**Affiliations:** 1 Department of Basic Medicine, Wuxi School of Medicine, Jiangnan University, Wuxi, 214122 Jiangsu P.R. China; 2 The School Hospital, Jiangnan University, Wuxi, 214122 Jiangsu P.R. China; 3 The Second People’s Hospital of Wuxi, Wuxi, 214002 Jiangsu P.R. China

**Keywords:** Alzheimer’s disease, Sleep, Three-dimensional reconstruction, Orexin A neurons, Amyloid-β

## Abstract

Sleep disturbance is common in patients with Alzheimer’s disease (AD), and orexin A is a pivotal neurotransmitter for bidirectionally regulating the amyloid-β (Aβ) deposition of AD brain and poor sleep. In the present study, we examined the characteristic of sleep–wake architecture in APPswe/PSldE9 (APP/PS1) and Aβ-treated mice using electroencephalogram (EEG) and electromyographic (EMG) analysis. We compared the expression of orexin A, distribution, and morphology of the corresponding orexin A-positive neurons using innovative methods including three-dimensional reconstruction and brain tissue clearing between wild type (WT) and APP/PS1 mice. Results from our study demonstrated that increased wakefulness and reduced NREM sleep were seen in APP/PS1 and Aβ treated mice, while the expression of orexin A was significantly upregulated. Higher density and distribution of orexin A-positive neurons were seen in APP/PS1 mice, with a location of 1.06 mm–2.30 mm away from the anterior fontanelle compared to 1.34 mm–2.18 mm away from the anterior fontanelle in WT mice. These results suggested that the population and distribution of orexin A may play an important role in the progression of AD.

**Supplementary Information:**

The online version contains supplementary material available at 10.1007/s00429-021-02447-w.

## Introduction

Alzheimer's disease (AD) is a progressive and irreversible neurodegenerative disorder characterized by diffused extracellular amyloid plaques deposition and intracellular neurofibrillary tangles (NFT) that results in progressive dementia associated with cognitive impairment, memory loss, and other behavioral abnormalities (Dey et al. 2017; Thal et al. 2019).

Sleep disturbances are commonly seen in patients with AD and affect approximately 25%–60% of patients (Lim et al. 2014). Compared with healthy older adults, individuals with AD suffer from shorter bouts of rapid eye movement (REM) sleep and more slow-wave sleep fragmentation (Vitiello and Prinz 1989; Mander et al. 2016). Insomnia and excessive daytime sleepiness were also common characteristics seen in AD patients (Roth and Brunton 2019; Hamuro et al. 2018).

Animal and human studies have demonstrated that the accumulation of the amyloid-β (Aβ) peptide, a primary cause of amyloid plaques, is a critical event in the pathogenesis of AD as well as poor sleep (Vanderheyden et al. 2018; Brown et al. 2016). Intracerebroventricular administration of Aβ has been identified to serve as a useful AD model which can trigger cognitive impairment, memory defects, and other AD-like alterations in the brain (Zhang et al. 2019b; Facchinetti et al. 2018). Many transgenic flies of AD models overexpressing Aβ peptides have shown significantly disrupted sleep–wake patterns including increased time awake and decreased sleep, including in transgenic APP and presenilin 1 (APP/PS1) mouse (Kent et al. 2018).

The finding that a single i.c.v. infusion of AβOs disrupted sleep pattern in mice proposed a direct regulation of Aβ on sleep disorder (Kincheski et al. 2017). However, the further mechanism which mediates this process is not clearly understood.

The orexinergic nervous system consists of two peptides: the orexin A/hypocretin-1 and orexin B/hypocretin-2, which are both synthesized by a cluster of neurons in the lateral hypothalamus and perifornical. These two orexins bind to two G-coupled protein receptors, i.e., orexin receptors 1 (OX1R, HCRTR-1) and 2 (OX2R, HCRTR-2), and participate in regulating the vital body functions, including sleep/wake architecture, food intake, cognition, and memory (Kukkonen et al. 2002; Thal et al. 2019; Burdakov 2019; Li and de Lecea 2020). A previous study demonstrated that orexin is primarily associated with interstitial Aβ level and wakefulness in transgenic AD mice (Kang et al. 2009). Orexin levels of cerebrospinal fluid (CSF) from AD patients were found to be higher than those seen in healthy people, and they are responsible for regulating wakefulness maintenance and prevent undesirable transitions into sleep (Liguori et al. 2016; Um and Lim 2020). Overexpression of orexins can lead to non-REM sleep fragmentation and REM sleep suppression during daytime (Willie et al. 2011; Makela et al. 2010). Orexin A, which has been shown to promote wakefulness, was recently highlighted on Aβ metabolism in animals and humans (Kang et al. 2009; Liguori et al. 2014). However, some studies carried out in humans have displayed conflicting conclusions. The activity of orexin A and its involvement in sleep/wake cycle alterations remain largely unknown, especially, in AD brain. Postmortem analysis revealed that the number of orexin-positive neurons in the hypothalamus and the concentration of orexin in ventricular CSF were reduced in patients with AD when compared with the controls (Fronczek et al. 2012). Some other studies demonstrated higher CSF orexin A levels in patients with AD when compared to the control group (Dauvilliers et al. 2014; Liguori et al. 2014; Wennstrom et al. 2012). This might be related to sleep deterioration and neurodegeneration (Liguori et al. 2014). Further evidence is needed to understand the link of orexin A to the underlying neurodegenerative process (Aβ deposition) or secondary to sleep/wake cycle alterations. Determining the morphology, distribution, and neural network for understanding the physiological function of orexin A neuron is essential for developing new clinical treatment strategies for poor sleep and AD. Therefore, the main objective of this study was to investigate sleep–wake features and the expression changes and distribution of orexin A underlying AD models.

## Materials and methods

### Animals

Male double transgenic APP/PS1 (APPswe/PSldE9) mice with C57Bl/6 J background aged 8 months were purchased from the Model Animal Center of Nanjing University of China (certificate No. 201501556; license No. SCXK (Su) 2015-001).

C57Bl/6 J mice were crossed to APP/PS1 mice to generate APP/PS1 and age-matched control Wild type (WT) mice. Mouse genotypes were determined by PCR. The following primers were used: APP: 5′- CTTGTAAGTTGGATTCTCATATCCG-3′, R: 5′- GACTGACCACTCGACCAGGTTCTG-3′; PS1: 5′- AATAGAGAACGGCAGGAGCA-3′, R: 5′-GCCATGAGGGCACTAATCAT-3′, the expected size of the PCR product is 344 bp for APP, 608 bp for PS1, and the product from littermates without the above target bands is used as WT control. All mice were housed in specific pathogen-free conditions under a 12 h:12 h light–dark cycle (lights on at 7 AM and lights off at 7 PM, illumination intensity ≈ 100 lx) at an ambient temperature of 22 ± 0.5 ℃ in the laboratory animal center of Jiangnan University.

The mice used in this study were approved by the Institutional Animal Care and Use Committee at Jiangnan University, Jiangsu, China.

### Reagent

Antibodies were purchased from several companies: Anti-c-Fos (Cat. No. sc-166940) from Santa Cruz (Santa Cruz, CA, USA); Anti-orexin A (Cat. No. ab6214) Abcam (Cambridge, MA, USA); Rhodamine Red-X-conjugated goat anti-rabbit/mouse IgG (Cat. No. BA1031; BA1032) from Boster Biological Technology (Wuhan, China); Aβ_1-42_ peptide (Cat. No. AS-65178) was purchased from AnaSpec (Bachem, CA, Switzerland); DAPI (Cat. No. C1006) was purchased from Beyotime, China.

### Experimental groups and animal treatments

Mice were randomly divided into four major groups (*n* = 20 for each group): WT (C57BL/6 J mice), APP/PS1, NS (WT + saline), and Aβ (WT + Aβ_1-42_).

Aβ(1–42) peptide was prepared according to the manufacturer instructions and previous publishment (Zheng et al. 2013). Briefly, Aβ1-42 peptide was dissolved in 1,1,1,3,3,3-hexafluoro-2-propanol (HFIP; Sigma-Aldrich) and aliquots were stored at − 20 °C. To generate soluble oligomers for injection, Aβ-HEIP peptide film was dissolved in dry dimethyl sulfoxide (DMSO) and incubated for 20 min, followed by the addition of sterile saline and a 20-min incubation in the HFIP/saline mixture. Subsequently, the solvent was incubated at 37 °C for 4 days before use. Oligomerization can be verified by dot blot assay as previously described (Cao et al. 2013).

Mice were anesthetized with intraperitoneal (i.p.) injection of chloral hydrate (350 mg/kg, i.p). A guide cannula (Ø = 0.5 mm, length = 15 mm) was stereotaxically implanted into the right lateral ventricle of the mice. The oligomerization of Aβ_1-42_ (410 pmol/mouse) was administered at the volume of 0.925 μL by intracerebral ventricular, through the planted guide cannula with the flow rate 1 μL/min. Saline was injected by intracerebral ventricular as control group of Aβ (WT + Aβ1-42). The coordinates of the guide tip were as follows: anteroposterior =  − 0.6 mm; mediolateral + 1.1 mm; and dorsoventral =  − 2.2 mm from bregma according to the atlases (Zheng et al. 2013). EEG recordings were performed following the injection of abeta or vehicle since 06:00. The samples of brain for IHC, c-Fos, and PCR assay were collected when sleep parameters’ recording was done 24 h since abeta administration. For the WT vs APP/PS1 group, mice were not injected saline, but compared directly.

### Polygraphic recordings and sleep–wake state analysis

For the sleep–wake cycle recording assay, four stainless steel screw cortical electrodes were screwed through the skull into frontal and parietal cortices to record electroencephalogram (EEG). The cortical electrodes were inserted into the dura through two pairs of holes located, respectively, in the frontal (1 mm lateral and anterior to the bregma) and parietal (1 mm lateral to the lambda) cortices. Three wire electrodes were directly inserted into the neck musculature for EMG recording. The ground electrode was placed on the skull over the cerebellum. Following the surgery, mice were housed in 12 h dark and 12 h light for 10 days. All mice were habituated to the recording cages for 3 days before starting the recording. The record was done since 06:00. After saline or Aβ administration for WT mice since 06:00, mice of each group were placed in a sound-attenuated, ventilated and electrically isolated chamber. EEG and EMG activities were amplified (2000) and filtered (0.5–60 Hz for EEG Model 3500, A-M Systems, WA, USA), and digitalized at a resolution of 256 and 128 Hz and recorded continuously with CED 1401 MKII (Cambridge Electronic Design Limited (CED), London, UK). The behavior of the mice during light and dark phases in the chamber was monitored and recorded using an infrared video camera. We visually scored polygraphic records by 30-s epochs for wakefulness (W), sleep (NREM), and REM according to previously described criteria validated for mice using a Spike 2 sleep-score script (CED) and with the assistance of spectral analysis by the fast Fourier transform (FFT) (Tsuneki et al. 2010; Harris et al. 2005).

### RNA isolation, reverse transcription, and quantitative PCR

After polygraphic recordings and sleep–wake states analysis were done, hypothalamus tissues of mice (10 were chosen from each group randomly) were collected and homogenized. Total RNA was extracted using TRIzol reagent (Corning, Shanghai, China). cDNA was synthesized using ReverTra Ace qPCR RT Kit (Toyobo, Osaka, Japan) and amplified by real-time PCR on a StepOne Plus system (Thermo Fisher Scientific, Waltham, MA, USA) with primer sets for Orexin A (forward, 5′- GCCTCAGACTTCTTGGGTATTT-3′; reverse, 5′- AGGGAACCTTTGTAGAAGGAAA -3′) and GAPDH (forward, 5′-TGCGACTTCAACAGCAACTC-3′; reverse, 5′-CTTGCTCAGTGTCCTTGCTG-3′). The relative expression (defined as fold change) of the target gene was given by 2^−△△Ct^ and normalized to GAPDH. At least triplicate independent experiments were performed.

### Immunohistochemistry of sequence sections

After polygraphic recordings and sleep–wake states analysis were done, 10 mice chosen randomly from each group were anesthetized with sodium pentobarbital (i.p. 80 mg/kg) and then sacrificed by intracardiac perfusion with cold phosphate-buffered saline (PBS) followed by 4% paraformaldehyde. After perfusion, the whole brain was removed, post-fixed in the same fixative for 2 days, and cryoprotected in 30% sucrose at 4℃ for 2 days. Brains were embedded in optimal cutting temperature compound (OCT) and cut on a freezing microtome (Leica CM1850; Leica Microsystems UK, Milton Keynes, UK) for acquiring coronal Sects. (30 μm) of the entire hypothalamus. All the sequence sections of these brains were processed for immunostaining. Each section was washed in PBS (3 × 5 min) and then processed 30 min in TritonX-100 in PBS, and blocked with 5% bovine serum albumin (BSA) and 0.2% TritonX-100 at room temperature for 1 h. The sections were then incubated with mouse anti-orexin A (1:1000) for 48 h on a shaker. The sections were washed thrice in PBS and were incubated with biotinylated goat anti-rabbit IgG. The brown chromogen deposition was shown with 3, 3′-diaminobenzidine tetrahydrochloride (DAB).

### Tissue optical clearing tracing and deep imaging

500-μm-thick coronal blocks of the brain were cut off and cleared using RapiClear 1.49 (SunJin Lab Co.) by immersing them overnight in the clearing reagent at room temperature. Cleared tissues were mounted on a custom-made sample holder.

Brain tissue from each group (10 mice/group) was dissected out and fixed in a 24-well plate with 4% paraformaldehyde solution on an orbital shaker for 2 h at room temperature. The samples were then transferred overnight into 2% PBST (2% Triton X-100 in PBS solution) for permeabilization. They were kept overnight in 10% BSA on an orbital shaker at 4 °C. The samples were then incubated with the primary antibody on an orbital shaker at 4 °C for 2 days after which they were incubated with secondary antibody at 4 °C for 1 day. After being washed with PBST, stain nuclear with DAPI, the imaged was acquired using the microscope (BX60, Olympus, Tokyo, Japan) and software of Imaris 9.3.0.

### Computer-assisted 3D reconstruction analysis

The distribution of stained cells was examined and reconstructed three-dimensionally. As described previously (George Paxinos et al. The Mouse Brain in Stereotaxic Coordinates), 130 slices including the hypothalamus that were at a distance of − 1.34 to − 2.18 mm from the bregma were reconstructed in each brain of WT mice. Around 150 slices including the hypothalamus which were at a distance of − 1.06 to − 2.30 mm from the bregma were reconstructed in the APP/PS1 mice. Operate a computer-assisted image processing and three-dimensional reconstructions using the ImageJ-win64 software, Adobe Photoshop CS4, and Amira 6.3.0.

### Statistic analysis

Data were expressed as mean ± SEM and analyzed using Prism 7 (GraphPad; San Diego, CA, USA). The differences among the two groups were performed using Student’s * t* test. Figure 2B, D is analyzed by two-way ANOVA. *P* value less than 0.05 was considered to be statistically significant in all the experiments.

## Result

### The sleep–wake architecture was disturbed in APP/PS1 and Aβ-injected mice

**Fig. 1:**
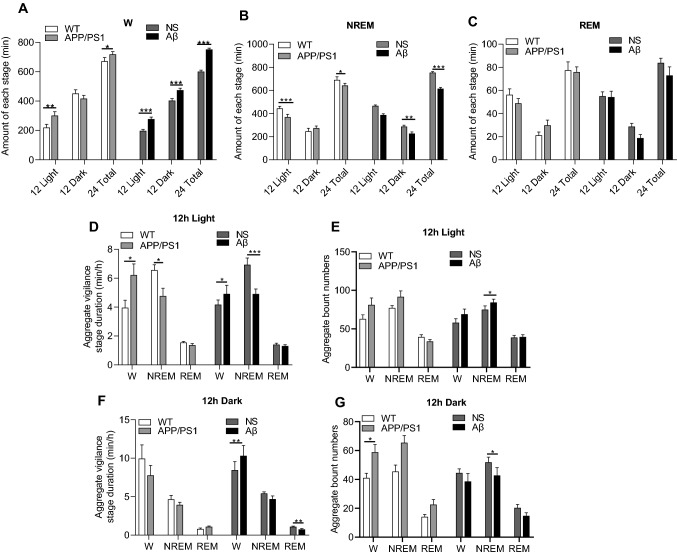
Sleep characteristics of WT, APP/PS1, saline, or Aβ-treated groups. Saline was injected by intracerebral ventricular as control group of Aβ, For the WT vs APP/PS1 group, mice were compared directly without saline injection. **A**–**C** Total time spent in W, NREM, and REM during the 12 h light, 12 h dark phases, and 24 h total was calculated; **D**–**G** aggregate vigilance stage duration and bount numbers for 12 h light and 12 h dark were calculated. Data expressed as means ± SEM (*n* = 6–8). *P < 0.05, **P < 0.01, *** P< 0.001. Data were analyzed using Student’s *t* test

We assessed the sleep state of APP/PS1 mice and Aβ-challenged mice to investigate the acute impact of Aβ on the sleep architecture. Compared to the control groups, APP/PS1 mice increased wakefulness by 42.9% in the 12-h light phase and 12.1% in 24 h total. Similarly, wakefulness was increased by 43.57% in the 12-h light phase, 17.62% in the 12-h dark phase, and 26.12% in 24-h total, respectively, in Aβ-treated mice (Fig. 1A).

The increase in wakefulness was concomitant with the reduction in non-rapid eye movements (NREM) sleep, and NREM sleep was decreased by 16.9% during 12-h dark period and 10.3% for 24-h total in APP/PS1 mice. In Aβ-treated mice, it was decreased by 18.41% in the 12-h light phase, 21.12% in the 12-h dark phase, and 19.62% in 24-h total (Fig. 1B).

However, no difference was observed in APP/PS1 or Aβ-treated mice in REM during the 12 h light, 12 h dark phases, or 24 h total when compared to the control group, respectively (Fig. 1C).

Compared to the control groups, the increase increased awakening time in the 12-h light phase of APP/PS1 mice was due to the increase of average duration, while the decrease of NREM was due to the decrease of average duration. There was no significant difference in the average duration and number of REM during the 12-h light and 12-h dark phases. Similarly, the increase of awakening caused by treatment with Aβ in the 12-h light phase was due to the increase of average duration, while the decrease of NREM was due to the decrease of average duration and the increase of occurrence number. The increase of awakening caused by treatment with Aβ in 12-h dark period was due to the increase of average duration. The decrease of NREM was due to the decrease of occurrence number. The average duration and number of REM in the 12-h light phase were not statistically significant. However, the average duration of REM decreased in the 12-h dark phase, while there was no significant change in the number of REM (Fig. 1D–G).

**Fig. 2:**
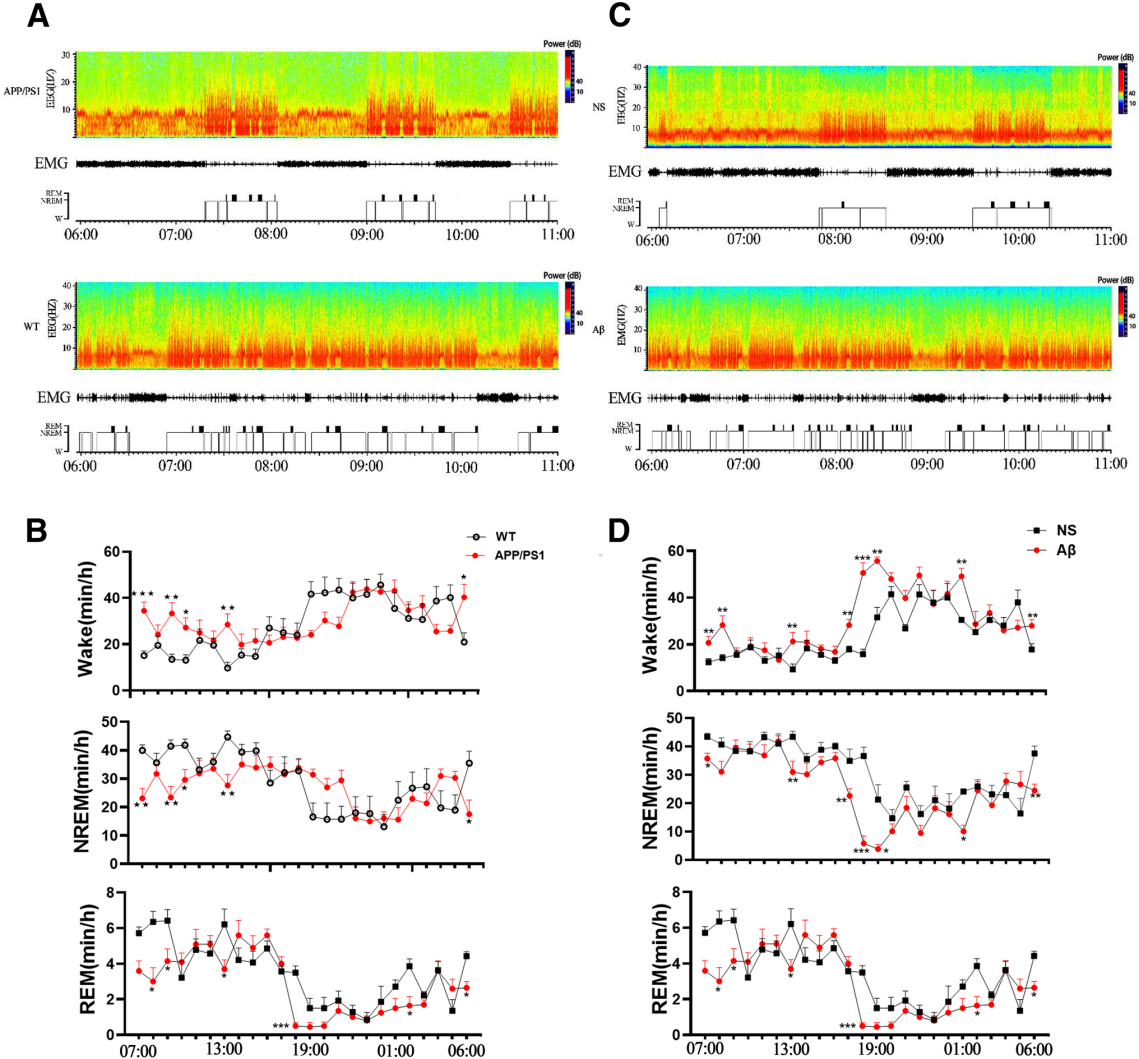
Sleep characteristics of WT, APP/PS1, saline, or Aβ-treated groups. **A** and **C** Representative spectrograms of EEG of brain state and EMG from 06:00 to 11:00 are shown. **B** and **D** The time-course changes of 24-h Wake, NREM, and REM. Data expressed as means ± SEM (*n* = 6–8). *P < 0.05, **P < 0.01, ***P < 0.001. Data were analyzed using by two-way ANOVA

An hour-by-hour analysis was carried out on the time-course from 24-h recordings. To begin with lights-on, time-course changes showed a significant increase in wakefulness, and this effect lasted for several hours. Specifically, the wakefulness of APP/PS1 mice increased at 07:00, 9:00–10:00, 13:00, and NREM decreased at the same time point in the light phase stage. At 19:00, 21:00, 5:00, and 6:00, APP/PS1 mice showed higher wakefulness in the dark phase, and at 19:00, 21:00, and 5:00 in dark phase. In REM, the decrease appears at 07:00 and 17:00 in the light phase, and 19:00, 21:00, and 5:00 in the dark phase (Fig. 2A, B). Increase in wakefulness at 17:00, 18:00, 19:00, 22:00, and 06:00, with a concomitant decrease in NREM at the same time. REM showed decreased at 18:00, 19:00, and 6:00 was seen in the mice treated with Aβ (Fig. 2C, D).

### The number of activated orexin A neurons and expression of orexin A were both upregulated in APP/PS1 and Aβ-injected mice

**Fig. 3:**
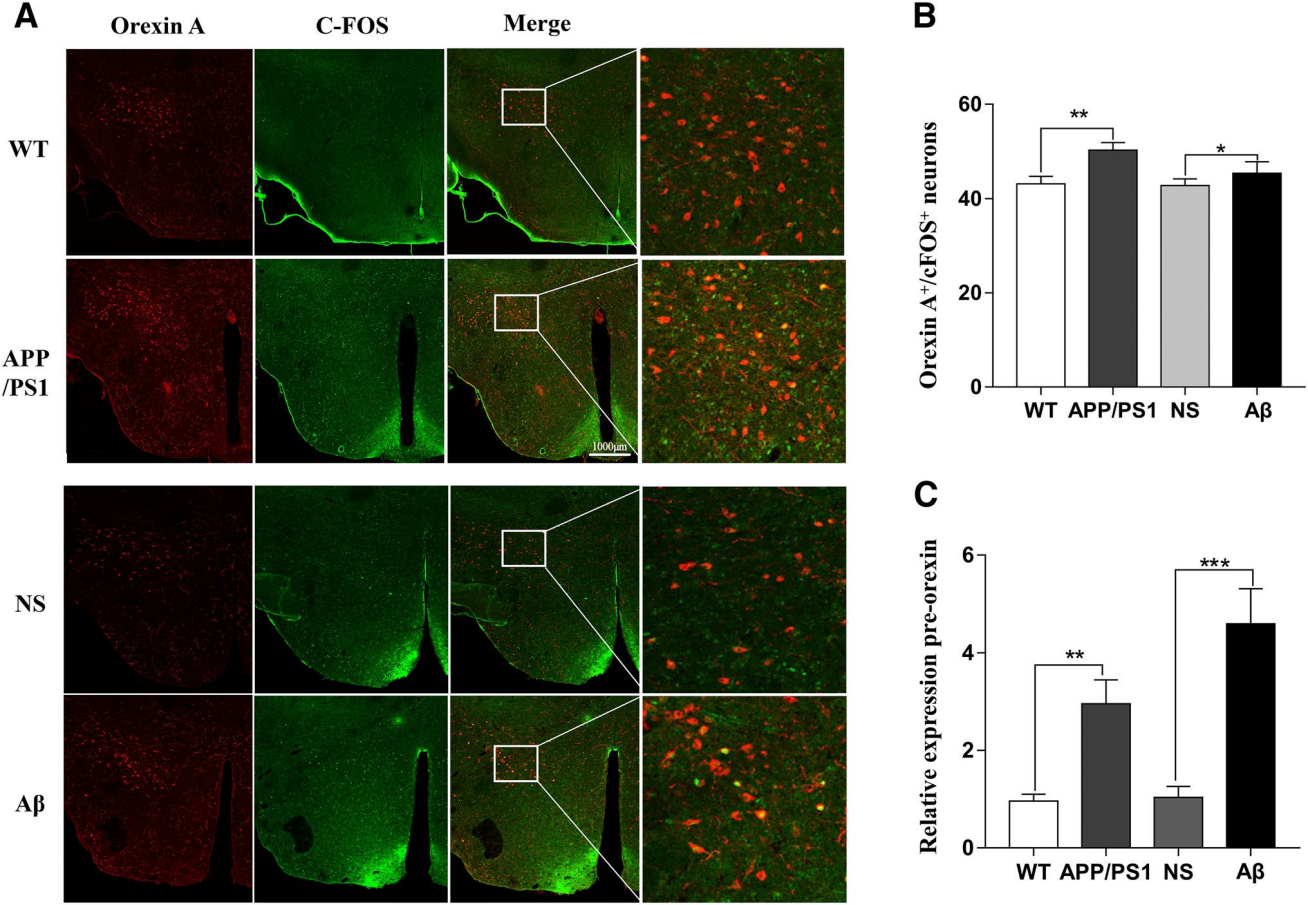
Orexin A neuronal activity and expression of orexin A were ascendant in APP/PS1, and Aβ-treated mice. **A** Immunofluorescence assay of orexin A and c-Fos in the different groups. **B** Quantitative analysis of c-Fos-positive orexin A neurons. **C** The prepro-orexin gene expression in the hypothalamus of different groups by quantitative real-time PCR. Data expressed as means ± SEM (*n* = 6–8). *P < 0.05, **P < 0.01, ***P < 0.001. Data were analyzed using Student’s *t * test

c-Fos gene, an immediate early gene that is transcribed when neurons are activated, has been extensively used as a marker of the activated neuron (Joo et al. 2016). We assessed the activated hypothalamic orexin neurons using immunofluorescence staining for c-Fos and orexin A. As shown in Fig. 3A, B, the number of activated orexin A neurons was significantly increased in APP/PS1 and Aβ-injected mice when compared with their relative control groups, respectively. Next, we examined the expression of orexin A and found that the mRNA levels of preproorexin, which is the common precursor of orexin A, were significantly upregulated in the APP/PS1 as well as the Aβ-injected mice (Fig. 3C).

### The density and distribution orexin A-positive neurons were increased in the brain of APP/PS1 mice

**Fig. 4:**
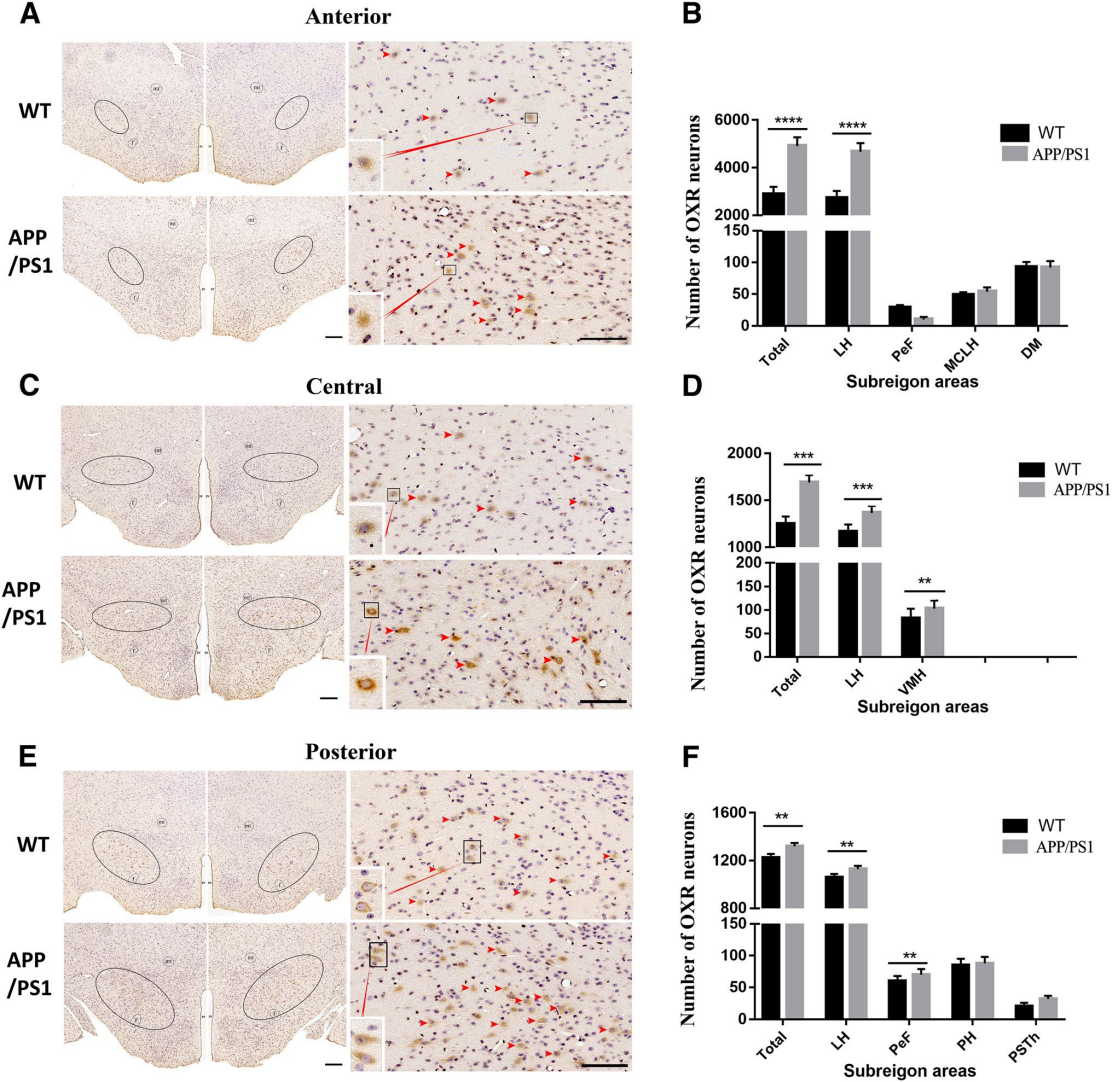
Orexin A-immunoreactive neurons in the anterior, central, and posterior sectors of the tuberal hypothalamus in coronal view. **A**, **B** The slices collected from the anterior sector were used for immunohistochemical staining for orexin A (brown) and quantitative analysis orexin A-positive neurons. **C**, **D** The slices from the central sector were used for immunohistochemical staining for orexin A (brown) and quantitative analysis orexin A-positive neurons. **E**, **F** The slices from the posterior sector were used for immunohistochemical staining for orexin A (brown) and quantitative analysis orexin A-positive neurons. LH lateral hypothalamus area; PSTH parabrain nucleus; PEF perifornix nucleus; PH posterior hypothalamic nucleus; VMH ventromedial hypothalamic nucleus; MCLH magnocellular nucleus of the lateral hypothalamus; dorsomedial hypothalamic nucleus. Scale bar: 100 μm. Data expressed as means ± SEM (*n* = 6–8). *P < 0.05, **P < 0.01, ***P < 0.001. Data were analyzed using Student’s *t* test

To demonstrate the rostral to caudal distribution of orexin A neurons in the brain of WT and AD mice, sequence coronal sections of different sectors were selected and analyzed using an immunohistochemistry assay. Orexin A positive neurons were distributed in an ellipsoid shape which was located in upper lateral of fornix, lower lateral of mammillothalamic tract, and symmetrically located on both sides of the third ventricle. The density of orexin A-positive neurons in the central sector of tuberal hypothalamus was much higher when compared to the anterior and posterior sectors (Fig. 4A, C, E). Compared to WT mice, the number of orexin A neurons totally and in HL and VMH in the anterior sector was significantly different (Fig. 4A, B). On the other hand, there was a statistically higher density of orexin A neuron totally and in LH of the central sector (Fig. 4C, D); and slightly decreased density of orexin A positive neurons totally and in LH as well as PeF in the posterior sector of tuberal hypothalamus in APP/PS1 mice when compared to the WT mice (Fig. 4E, F). The total density and distribution range of orexin A positive neurons were significantly higher in the brain of APP/PS1 mice compared to that of WT mice.

### Three-dimensional reconstruction showed increased orexin A neurons in the brain of AD mice

**Fig. 5:**
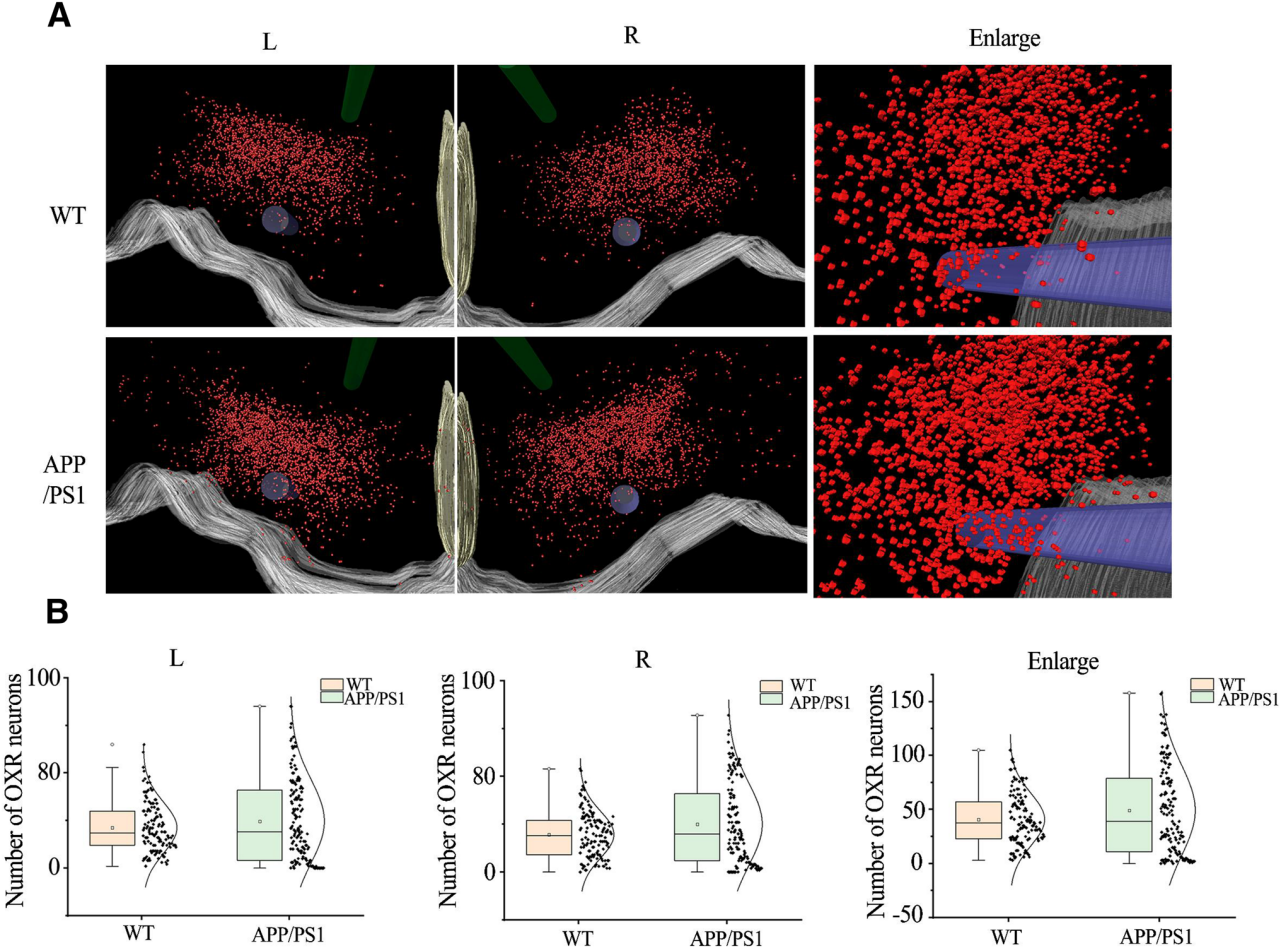
Distribution of orexin A-immunoreactive neurons in the WT and APP/PS1 mouse. **A** Orexin A neurons on both sides of the WT and AD mice. Each bright red dot represents a single orexin-immunoreactive neuron. Other three-dimensional structures are as follows: pale yellow—third ventricle; light green—mammillothalamic tract; violet—fornix; gray—ventral floor. **B** The Box plot and normal curve demonstrate the distribution of the total number of ORX-ir neurons in the WT and APP/PS1 mice. The median, lower and upper quartile, an extreme case, the outlier was estimated using the Box plot

Three-dimensional reconstruction was conducted based on the immunohistochemistry image of orexin A-positive neurons. Orexin A-positive neurons were mainly located in the tubercular hypothalamic region, and were accompanied with fornix and mammillothalamic tract in both WT and APP/PS1 mice. The distribution and density of orexin A-positive neurons in APP/PS1 mice were higher in APP/PS1 mice when compared with WT mice (Fig. 5A, B). These results indicated that orexin A-positive neurons were higher in the brain of AD mice when compared with WT mice.

### Maps accurately showed the distribution of orexin A-positive neurons

**Fig. 6:**
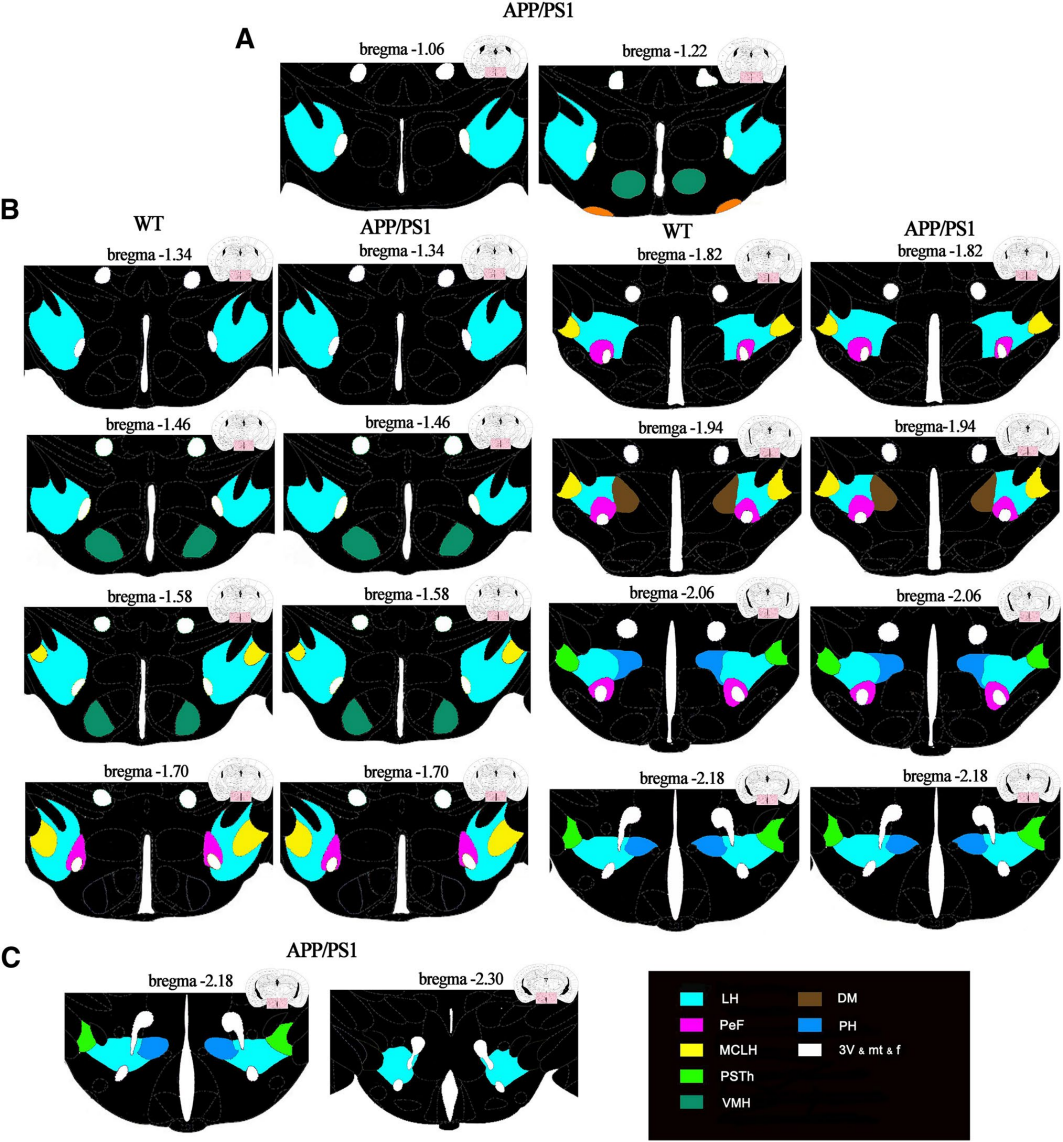
Sections are ordered from rostral to caudal, the hypothalamus was inconspicuously subdivided by the splitting dotted line; schematic drawings showing the general morphology of the nucleus orexin A-immunoreactive distributed in the **A** ORX-ir neurons were shown from distance of − 1.06 mm and − 1.22 mm away from the bregma (the reference zero point) in APP/PS1 mice. **B** ORX-ir neurons were shown from distance of -1.34 mm to -2.18 mm away from the bregma. **A**, **C** ORX-ir neurons were shown from distance of − 2.18 mm and − 2.30 mm away from the bregma in APP/PS1 mice (6 mice/group)

It has been identified that orexin A-positive neurons were mainly located in the tuberal hypothalamus (Peyron et al. 1998), and we further analyzed the detailed distribution of orexin A positive neurons using the serial slices from the rostral to caudal of WT and APP/PS1 mice. The results demonstrated that orexin A-positive neurons were seen at a distance of 1.06 mm from the bregma that in the APP/PS1 and their number was higher once the distance from the bregma was increased. Most orexin A-positive neurons were seen in the lateral hypothalamus area (LH). A few were seen in the ventromedial hypothalamic nucleus (VMH) and supraoptic nucleus (SOR) in 1.22 mm far away from the bregma, while there has no orexin A positive neurons until this location in WT mice (Fig. 6A). The range of orexin A-positive neurons in APP/PS1 mice was still limited in the area of LH, VMH, and SOR even the number of orexin A neurons increased in 13.4 mm far away from the bregma, where several orexin A neurons were found in LH of the brain of WT mice. Then, the distribution location changed as the distance increased. Till a distance of 2.06 mm from the bregma, the orexin A neurons in WT mice were mainly found in the parabrain nucleus (PSTH), perifornix nucleus (PEF), and posterior hypothalamic nucleus (PH). Orexin A positive neurons in the APP/PS1 mice displayed a broader range, locating in another region of medial tuberal nucleus (Mtu) except for PSTH, PEF, and PH (Fig. 6B). At a distance of 2.30 mm from the bregma, orexin A-positive neurons in the brain of APP/PS1 mice were found only in LH, while no orexin A positive neurons were seen in the brain of WT mice at the same distance (Fig. 6C). Taken together, the above data suggested that the orexin A-positive neurons appeared earlier, but disappeared later from the rostral to caudal of brain in APP/PS1 mice when compared to WT mice.

### RapiClear cleared the brain of WT and APP/PS1 mice

**Fig. 7:**
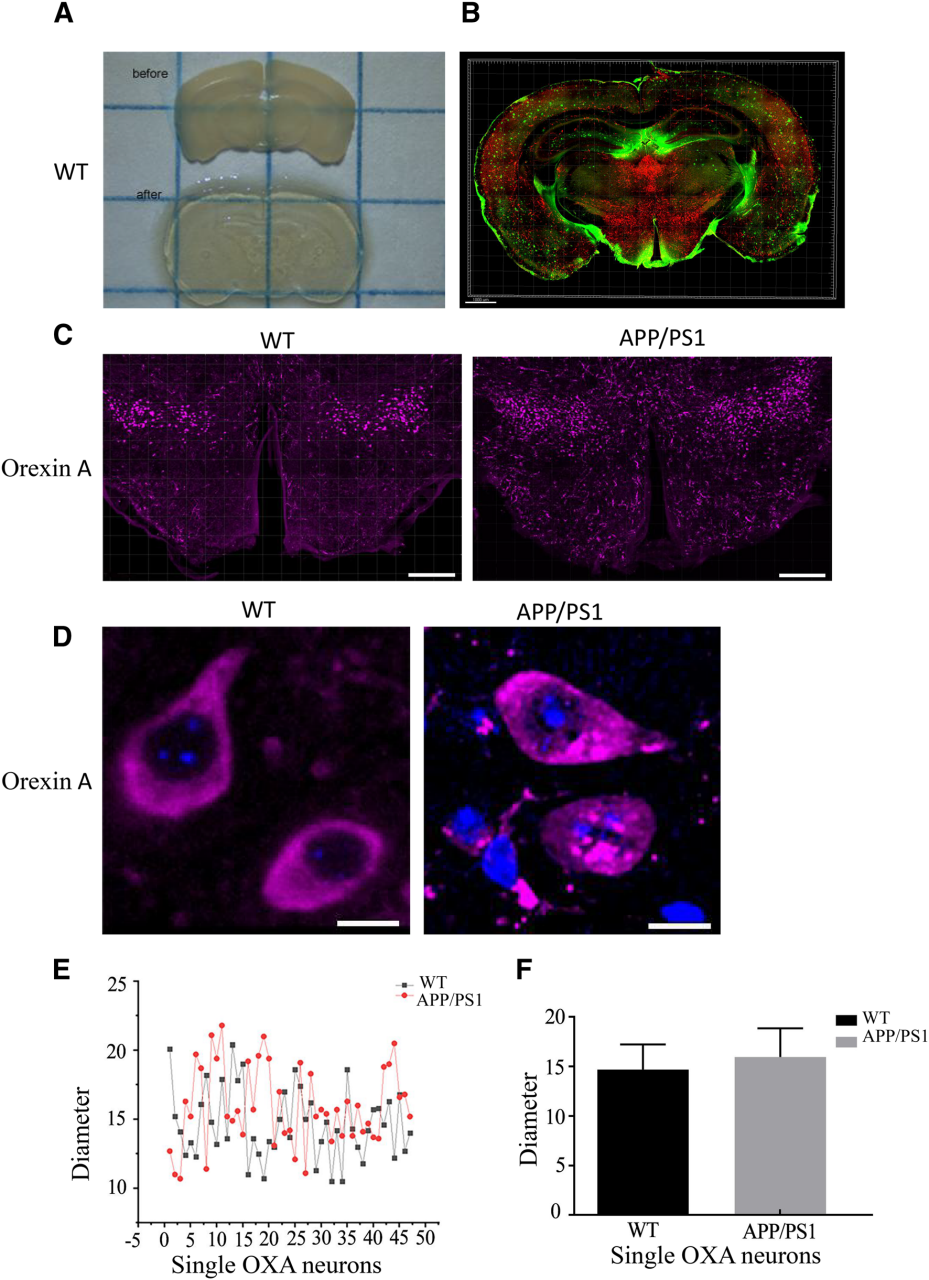
RapiClear cleared mouse brain. **A** The change of mouse brain after optical clearing. **B** Immunofluorescent staining against OXA-ir (red) and anti-β-Amyloid (green) antibody in the whole RapiClear cleared brain. **C**, **D** Immunofluorescent staining and magnification for orexin A neurons (purple); DAPI staining was to show the nuclear (blue). Scale bars: 100 μm. **E** Linear graph shows the diameter of a single cell of WT and APP/PS1 mice (*Y*-axis units: micrometer). **F** Quantitative analysis of the diameter for orexin A neurons

Generally, the serial slice may arise some deformation and damage of tissues, and inaccuracy may occur during the three-dimensional reconstruction. Thus, we adopted the RapiClear technique to further examine the distribution of orexin A positive neurons, which can keep the tissue structure to analyze the three-dimensional and topological morphology of neurons. As shown in Fig. 7A, the brain tissue becomes uniformly transparent after immersion in refractive-index-specified solutions.

The tissue structure, cellular architecture, and fluorescence signals were preserved well in RapiClear cleared brain (Fig. 7B). It was also shown that the densities of orexin A neurons in the brain of APP/PS1 mice were significantly higher compared to WT mice (Fig. 7C). We then observed the morphology of orexin A neurons, and found that the diameter of these neurons was always around 10–25 μm and did not show any significant difference between the brains of WT and APP/PS1 mice (Fig. 7D-F).

## Discussion

Sleep abnormalities have been observed for decades in AD (Weng et al. 2020). In the present study, we examined the sleep–wake cycle and evaluated the relationship between immunoreactivity and expression of orexin A and Aβ in the tuberal hypothalamus with a special focus on the spatial distribution of labeled neurons of AD models.

Sleep dysfunction is considered a core component of AD. 6-month-old APP/PS1 AD model has previously been shown to exhibit increased wakefulness during the 12 h light phase (Zhang et al. 2019a; Zhurakovskaya et al. 2019); some other studies have reported that 9-month-old APP/PS1 female mice displayed reduced REM and NREM sleep stages across both light and dark phases (Roh et al. 2012); reduced NREM and increased wakefulness during 12 h light phase also has been observed in PLB1 Triple (Platt et al. 2011). There is mounting evidence that Aβ amyloidosis plays a key role in the bi-directional regulation of AD pathology in the brain and sleep disorder (Boespflug and Iliff 2018). In addition, reduced NREM sleep was also reported to be associated with high cerebrospinal fluid Aβ42 levels in cognitively control elderly subjects (Varga et al. 2016). Sleep-weak episode number and mean duration have not been previously performed, though several studies explored sleep disorder using EEG/EMG in transgenic AD mice. Results from our study demonstrated increased wakefulness with ascendant mean duration and decreased sleep in the time of lights-on and light-off translation in APP/PS1 mice using sleep episode and duration analysis.

The impact of AβOs on sleep time in mice has previously been identified by ICV injection of Aβ oligomers, showing that mice infused 100 pmol AβOs spent decreased sleep time between 5 and 8 days post-injection (Kincheski et al. 2017). Our current study also revealed that Aβ-administrated mice spent excessive awakening and less NREM during the light and dark phase since injection, and using EEG measurements, we futher made direct claims concerning the effects of AβOs on duration or frequency of sleep stages and providing direct evidence that the accumulation of Aβ is a crucial factor to promote wakefulness in the progression of AD. The sleep–wake cycle is accurately regulated by many brain areas and neural circuits. This includes the brainstem, midbrain, thalamus, hypothalamus, and basal forebrain. Recently, the orexinergic system is receiving extensive attention in AD for its vital function. High levels of orexin in the cerebrospinal fluid with sleep impairment were seen in patients with AD (Gabelle et al. 2017), (Liguori et al. 2014). Some studies have demonstrated that blocking of the orexinergic system might significantly contribute to a reduced level of Aβ and subsequent awakening (Hagan et al. 1999; Lee et al. 2005). Treatment of sleep disorder has been identified to be an effective strategy for improving pathological changes seen in AD patients with poor sleep (Cousins et al. 2019). Our results demonstrated that the amount of activated orexin A neurons labeled with c-Fos were upregulated, accompanied by enhanced expression of preproorexin in APP/PS1 and Aβ-treated mice.

Based on these alterations of physiology and biologic level, we established the anatomical display for spatial distribution, cell density, and cellular morphology of orexin A neurons from the three-dimensional structure. The data demonstrated that neurons immunoreactive to orexin A were distributed in an ellipsoid shape centered in the tuberal hypothalamus and located dorsally to the fornix, with fewer neurons in the anterior and posterior sectors. Central sectors of the brain displayed a higher population of orexin A-positive neurons, which were observed with no significant increase or even small decrease in anterior and posterior sectors of the brain when APP/PS1 mice were compared to the WT mice. This phenomenon may be formed of extended area orexin A neurons in APP/PS1 mice leading to a relative lower average subpopulation of each slice in the corresponding sectors, with a location of 1.06 mm to 2.30 mm away from the anterior fontanelle in APP/PS1 mice compared to 1.34 mm to 2.18 mm away from the anterior fontanelle in WT mice. It has been well established that orexin A-positive neurons in LH can regulate sleep–wake cycle behavior via several neural circuits. Orexin receptors exist in monoaminergic neurons, including noradrenaline (NE) neurons in LC, histamine (HA) neurons in tuberomammillary nucleus (TMN), and 5-hydroxytryptamine (5-HT) neurons in dorsal raphe nucleus (DRN), which enables orexin neurons to project to them and maintain wakefulness (Mieda et al. 2011); Second, orexin neurons in the LH region also receive projections from sleep-related nuclei. GABAergic neurons in the ventrolateral preoptic nucleus (VLPO) and median preoptic area (mnpo) densely project to orexin neurons in the LH region. Light stimulation of GABAergic neurons in these two nuclei during sleep can effectively inhibit orexin neurons in the LH region, to maintain sleep state (Saito et al. 2013). Here, in our study, the increase and earlier appearance of distribution and orexin A positive neurons may explain the sleep disorder in AD mice. Additionally, in the AD model, a few orexin A neurons exist in the region of the periventricular nucleus (PE), the margin of the third ventricle. These neurons might contribute to the progression of AD via activation of related mediator-regulated signal protein in CSF of the third ventricle.

The structure, distribution, and projection characteristics of orexin A neurons are the basis for various physiological functions, which determine the properties of synaptic bioelectrical signals and the ability to transmit information. It was reported that there is a functional dichotomy for orexin neurons. Those which are located in perifornical and dorsomedial hypothalamic areas (PFA–DMH) regulate arousal, waking, and response to stress, while those which are present in the lateral hypothalamus and ventral tegmental area are involved in reward-based learning and memory (Harris and Aston-Jones 2006). Using a dual retrograde tracer strategy, another study proposed that orexin neurons can be classified based on their downstream projections, whereas these classifications do not show a topographic location within the hypothalamus. Orexin neurons projected to the LC and TMN were mainly involved in wakefulness/arousal populations), and those projected to NAc and VTA regulate reward populations (Iyer et al. 2018). A few results were seen for the structure and distribution of orexin A neurons shown from three-dimensional morphology, which was just limited in two-dimensional space (Luna et al. 2017; Cheng et al. 2003). In the current study, we compared the expression, population, and morphology by different experiments including tissue optical clearing tracing, deep imaging, and three-dimensional reconstruction, determining increased population and more extensive distribution in the AD model. Orexin neurons in some regions are indistinct and their function in the regulation of sleep and progression of AD needs to be explored further. Additionally, previous studies have shown that orexin A neurons could be both single and bipolar cells with round, oval, and spindle shapes, exhibiting an average cell diameter of about 21 μm in the brain of rats (Cheng et al. 2003). We found that the shape of orexin was also diversified in mouse brains with a diameter of 10–25 which was slightly smaller than that of rats.

There is increasing evidence which indicates that the orexin system is strongly implicated in sleep disorder and AD pathogenesis (Kang et al. 2009; Liguori et al. 2020), (Kang et al. 2009; Liguori et al. 2020). Future studies should investigate the possibility of potential therapeutic strategy by direct pharmacological intervention at the differentially distributed orexin A neurons in patients with AD.

## Supplementary Information

Below is the link to the electronic supplementary material.

Supplementary file1 (DOCX 27 KB)

## Data Availability

The data, material, and code that support the findings of this study are available from the corresponding author [HongXu, Sun], upon reasonable request.
