# Supplementary material for: Orexin A peptidergic system: comparative sleep behavior, morphology and population in brains between wild type and Alzheimer’s disease mice
Source: Brain Struct Funct. 2022 Jan 23;227(3):1051–65. doi: 10.1007/s00429-021-02447-w (PMC8930968; doi:10.1007/s00429-021-02447-w)
Supplement: Supplementary file 1 — Supplementary file1 (DOCX 27 KB) [file 429_2021_2447_MOESM1_ESM.docx]

Table 1. The P values of the statistical analyses in our Figures。
